# A Treatise on the Use of Adhesive Gold Foil

**Published:** 1857-10

**Authors:** Robert Arthur


*A Treatise on the Use of Adhesive Gold Foil.*—By Robert Arthur. This is the title of a work of 86 pages, recently published by Jones, White & McCurdy, of Philadelphia. It not only gives the result of the experience of the author, but also explicit directions for the preparation and use of the gold. In the use of adhesive foil, an entirely different system of manipulation, from that employed in filling teeth with ordinary foil, is required, and as there are many cases in which a better operation can be made with gold of this description, than with common foil, the present treatise cannot prove otherwise than very serviceable to the profession. It constitutes a valuable acquisition to the literature of operative dentistry, and we take great pleasure in directing the attention of our readers to it. The author, recently a professor in the Philadelphia College of Dental Surgery, is already well known to the profession, as a highly accomplished practitioner.

We regret that in the hurry of getting the present number of the Journal from the press, we have not time to give an analysis of this excellent treatise, or to give the result of our own experience in the use of adhesive foil, as every effort on the part of individual members of our profession to advance this as well as other branches of practice, is worthy of the highest praise, and notwithstanding the great improvements that have been made within the last few years in mechanical dentistry, the surgical or operative department has progressed with equal rapidity. Diseased teeth are now daily restored to health and rendered useful, which, twenty years ago, would have been thought beyond the reach of the skill of the dentist, and we have no idea that the *ne plus ultra* of perfection in the treatment of the morbid conditions of these organs has even yet been attained.

In the preparation of the work before us, Dr. Arthur has shown himself to be well acquainted with the subject on which he has written, and that it will be productive of much good, we have no doubt. The favorable opinion which we entertain of it, enables us, therefore, to recommend it in the most unqualified manner to our professional brethren.

